# Impaired Elastin Deposition in *Fstl1^−/−^* Lung Allograft under the Renal Capsule

**DOI:** 10.1371/journal.pone.0081368

**Published:** 2013-11-25

**Authors:** Yan Geng, Lian Li, Yingying Dong, Xue Liu, Xiao-He Li, Wen Ning

**Affiliations:** 1 College of Life Sciences, Nankai University, Tianjin, China; 2 School of Pharmaceutical Science, Jiangnan University, Wuxi, China; Children's Hospital Los Angeles, United States of America

## Abstract

Lung alveolar development in late gestation is a process important to postnatal survival. Follistatin-like 1 (Fstl1) is a matricellular protein of the Bmp antagonist class, which is involved in the differentiation/maturation of alveolar epithelial cells during saccular stage of lung development. This study investigates the role of Fstl1 on elastin deposition in mesenchyme and subsequent secondary septation in the late gestation stage of terminal saccular formation. To this aim, we modified the renal capsule allograft model for lung organ culture by grafting diced E15.5 distal lung underneath the renal capsule of syngeneic host and cultured up to 7 days. The saccular development of the diced lung allografts, as indicated by the morphology, epithelial and vascular developments, occurred in a manner similar to that in utero. Fstl1 deficiency caused atelectatic phenotype companied by impaired epithelial differentiation in D3 *Fstl1^−/−^* lung allografts, which is similar to that of E18.5 *Fstl1^−/−^* lungs, supporting the role of Fstl1 during saccular stage. Inhibition of Bmp signaling by intraperitoneal injection of dorsomorphin in the host mice rescued the pulmonary atelectasis of D3 *Fstl1^−/−^* allografts. Furthermore, a marked reduction in elastin expression and deposition was observed in walls of air sacs of E18.5 *Fstl1^−/−^* lungs and at the tips of the developing alveolar septae of D7 *Fstl1^−/−^* allografts. Thus, in addition to its role on alveolar epithelium, Fstl1 is crucial for elastin expression and deposition in mesenchyme during lung alveologenesis. Our data demonstrates that the modified renal capsule allograft model for lung organ culture is a robust and efficient technique to increase our understanding of saccular stage of lung development.

## Introduction

The mammalian respiratory system fulfills multiple functions related to terrestrial living and breathing air [1]. In the mouse embryo, lung development begins at embryonic day 9 (E9), two buds arise from the ventral foregut endoderm and undergo stereotypic branching to form the embryonic lung during the pseudoglandular stage (E9.5–E16.5). At around E16.5 in the mouse, lung development switches from branching morphogenesis to a process of alveologenesis, including canalicular (E16.5–E17.5), saccular (E17.5–P5) and the final alveolar (P5–P30) stages, at which time the embryonic lung matures into an efficient gas-exchange unit by developing numerous alveoli [1], [2]. The formation of alveoli is characterized by the ingrowth of ridges or crests known as secondary septae that subdivide the terminal air sacs into alveoli. This process requires the migration of alveolar myofibroblasts into these ridges and the deposition of elastin at the tips of developing septae [3]. Several signaling factors, including Pdgfα [4], Ephrin B2 [5], Fgfr3/Fgfr4 [6] and RARb [7], have been shown to be especially important for alveologenesis by the lung phenotype of their genetic deficient mice [1]. However, the precise mechanism underlying alveologenesis is largely unclear.

*Fstl1*, first identified as a TGF-β1-inducible gene in a murine osteoblastic cell line (MC3T3-E1) [8], encodes an extracellular glycoprotein whose amino acid sequence has similarity with follistatin and the secreted protein rich in cysteine (SPARC) [9], [10]. FSTL1 has been shown to serve as a tumor suppressor gene [11], [12], act as a cardioprotective factor [13], [14], [15], act as a key molecule in rheumatoid arthritis [16], [17], [18], [19] and serve as one of the endogenous TLR4 agonists [20]. Fstl1 is highly conserved in vertebrates and plays an important role in vertebrate development [21]. Early loss of *Fstl1* in chick, zebrafish or frog is associated with defects in both establishment of the dorsoventral body axis and decreased neurulation [22], [23], [24]. Target-deletion of *Fstl1* in mice results in multiple developmental defects, including lung [25], skeleton [26] and ureter [27]. We have previously generated *Fstl1* deficient mice and reported the important role of Fstl1 on the differentiation/maturation of alveolar epithelial cells (AECs), by negatively regulating Bmp4 signaling during saccular stage of lung development [25]. Further study of Fstl1 on the alveolar formation in the late alveologenesis has not been pursued because of the postnatal death of *Fstl1^−/−^* pups and the lack of *ex vivo* organ culture models.

The study of late stages of lung development has relied primarily on the transgenic and gene targeting mouse models. Further *in vitro* culture models are badly needed to increase our understanding of the molecular basis underlying lung alveolar development. Whole fetal lung organ culture is a useful model for lung branching morphogenesis [28], [29], but not for alveologenesis due to the lack of a blood supply *in vitro*. The blood vessels which have developed from the beginning in parallel with the airway have been proved to be critical for alveolar formation during alveologenesis. Comparatively, whole fetal lung allograft under the renal capsule provides a suitable *in vitro* model for alveologenesis, because vessels develop in lung allografts and also connect to the vasculature of the host mice [30].

In this study, we adopted the renal capsule allograft model and modified it by grafting the diced E15.5 distal lung underneath the renal capsule of syngeneic mice. We found that the saccular development of these diced lung allografts occurs in a manner similar to that in utero. With the help of this *ex vivo* organ culture model, we further reported that Fstl1 is essential for elastin production and alveolar septation.

## Results

### Gross morphology of diced lung allografts under the renal capsule

**Figure 1:**
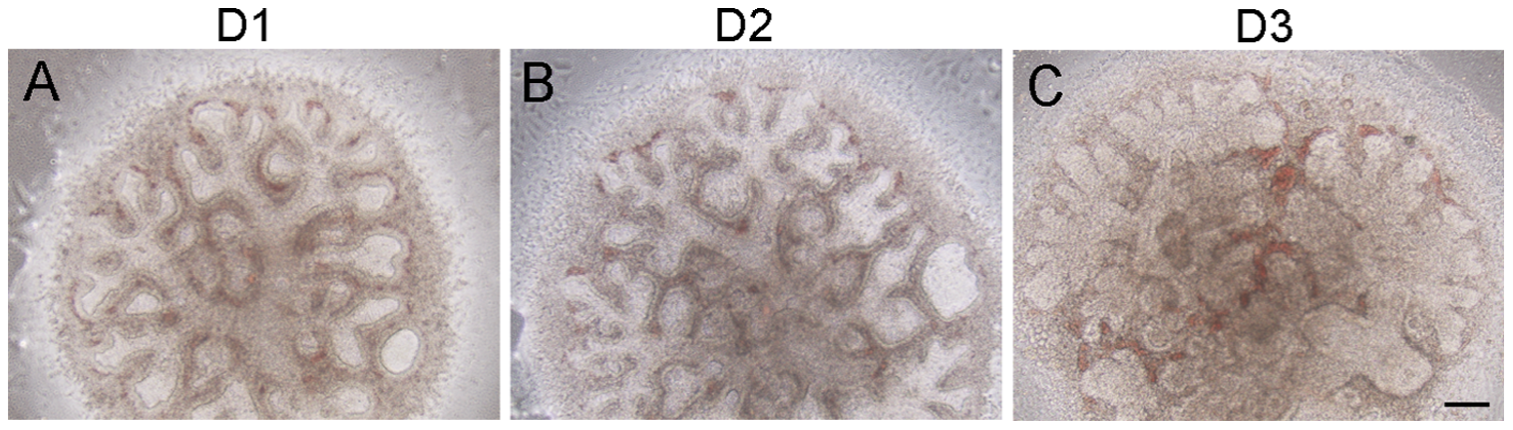
Morphogenesis of murine fetal distal lung explants in organ cultures. E15.5 WT lungs were diced and *in vitro* cultured. Microscopic analysis of explants at indicated time points. (A) day 1, (B) day 2 and (C) day 3. Scale bar, 100 μm.

**Figure 2:**
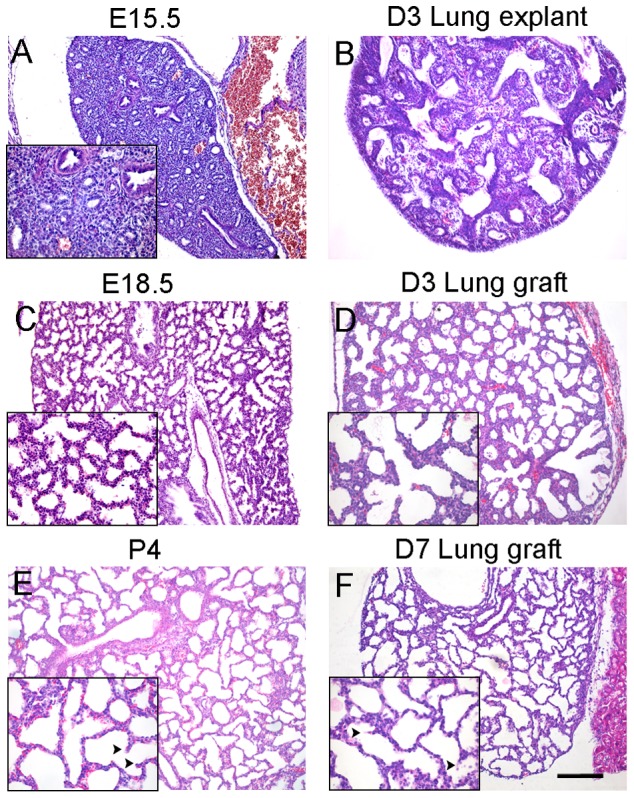
Morphogenesis of murine fetal distal lung allografts underneath the renal capsule. E15.5 WT lungs were diced and implanted underneath the renal capsule. Sections of fetal lung tissues, explants and allografts at indicated time points were stained with Hematoxylin and eosin (H&E) and shown at low and high (inserts) magnification. (A) E15.5 lung tissue, (C) E18.5 lung tissue, (E) P4 lung tissue, (B) day 3 explant, (D) day 3 allograft, (F) day 7 allograft. Arrowheads indicate secondary septae (E–F). Scale bar, 100 μm.

We previously used E15.5 lung explants to study the role of Fstl1 on lung saccular maturation [25]. The *in vitro* cultured E15.5 wild type (WT) explants displayed a modest increase in size combined with dilation of the terminal airway tubes (Figure 1A–C) and could only survive for 3 days in maximum. Sections of D3 lung explants contained the main bronchi surrounded by abundant mesenchyme and a few further branches, phenotype similar to that of E15.5 lungs *in vivo*, but not E18.5 lungs (Figure 2A–C), suggesting that the terminal airway branches fail to develop in cultured E15.5 lung explants. Thus, E15.5 lung explant culture is not a good *in vitro* model for the study of alveologenesis.

To facilitate the functional study of Fstl1 in the late gestation stage of terminal saccular formation, we adopted the renal capsule allograft model of lung organ culture [30]. Instead of using the E12.5 whole lung [30] or high-density mixed cell aggregates from E14.5 lungs [31] as the graft underneath the renal capsule of immunocompromised mice, we diced E15.5 WT whole fetal lung into small blocks (0.5 to 1.0 mm^3^) and grafted them underneath the renal capsule of syngeneic mice (FVB/N background). The diced lung allografts were harvested by 3 days or 7 days and subjected for morphological analysis. Compared to D3 lung explants, sections of D3 lung allografts displayed many small distal air sacs, a typical phenotype of E18.5 lungs in utero (Figure 2C–D), indicating the continuous saccular development in allografts. After 7 days under the renal capsule, lung allografts developed further with tremendous increase in size and substantial thinning of sac walls (also known as the primary septae) (Figure 2E–F). Histological examination of D7 lung allografts also showed the ingrowth of ridges or crests known as secondary septae (Figure 2E–F, arrowheads in high magnification views), findings consistent with the morphology of postnatal day 4 (P4) lungs. When the diced lung allografts were harvested after 14 days, no further secondary septation, which is typical in P8-10 lung, was observed (data not shown). Our data suggest that the diced allografts of late pseudoglandular lung are viable under the renal capsule of syngeneic host and continue to develop into saccular stage without significant morphological delay.

### Cell differentiation in diced lung allografts under the renal capsule

**Figure 3:**
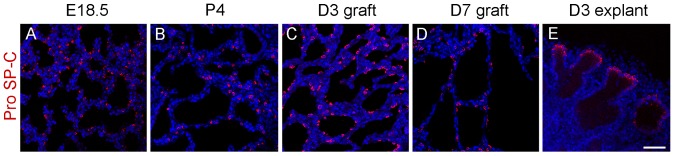
Differentiation of AEC-II cells in lung allografts. E15.5 WT lungs were diced and implanted underneath the renal capsule. Sections of fetal lung tissues, allografts and explants at indicated time points were immunostained with pro SP-C antibody. (A) E18.5 lung tissue, (B) P4 lung tissue, (C) day 3 allograft, (D) day 7 allograft, (E) day 3 explant. Pro SP-C-positive AEC-II cells were found scattered in saccular structures of fetal lung tissues and renal capsule allografts (A–D), while, pro SP-C-positive AEC-II cells were found lining the distal acinar tubules in lung explants (E). Red color: pro SP-C; blue color: DAPI. Scale bar, 50 μm.

**Figure 4:**
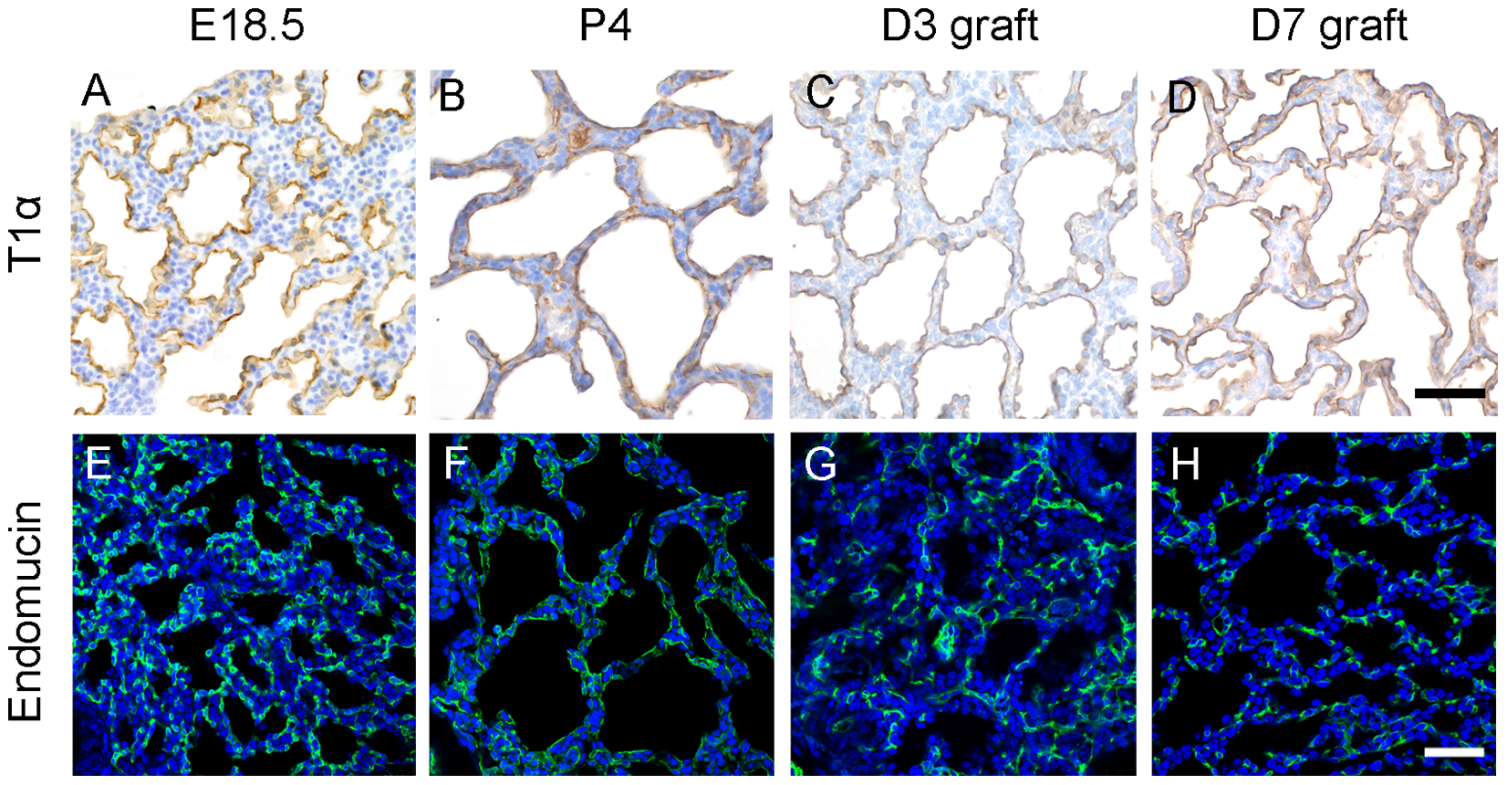
Differentiation of AEC-I cells and blood vessel development in lung allografts. E15.5 WT lungs were diced and implanted underneath the renal capsule. Sections of fetal lung tissues and allografts at indicated time points were immunostained with T1α or Endomucin antibodies. (A) and (E) E18.5 lung tissues, (B) and (F) P4 lung tissues, (C) and (G) day 3 allografts, (D) and (H) day 7 allografts. T1α-positive AEC-I cells were found lining the saccular lumen of fetal lung tissues and renal capsule allografts (A–D). Endomucin-positive endothelial cells were found next to the saccular epithelium of fetal lung tissues and renal capsule allografts (E–H). Green color: Endomucin; blue color: DAPI. Scale bar, 50 μm.

At E18.5 to P4, the peripheral lung consisted of saccules lined by both squamous type I cells (AEC-I) and cuboidal type II cells (AEC-II), indicating structural maturation typically at this stage of gestation [1]. To test whether the epithelial differentiation occurred properly in diced lung allografts under the renal capsule, markers for AEC-II cells (pro SP-C) and AEC-I cells (T1α) were used to immunostain sections of D3 or D7 allografts. Consistent with the WT E18.5 and P4 lungs, pro SP-C staining marked the scattered distribution of AEC-II cells and T1α staining labeled the continuous AEC-I cells on the apical surface of the saccular walls in D3 or D7 lung allografts (Figure 3A–D and 4A–D). This is in contrast to D3 lung explants, where the peripheral pro SP-C staining indicated the arrest of the epithelial development in the pseudoglandular stage (Figure 3E).

At this stage, the capillaries also grow rapidly in the mesenchyme surrounding the saccules to form a complex network. To assay the vascular formation in lung allografts, sections of E18.5 lung and lung allografts were immunostained with Endomucin, an endothelial cell marker [32]. As shown in Figure 4, there was a network of blood vessels in the mesenchyme surrounding the saccular walls (Figure 4G–H) of D3 and D7 lung allografts, findings similar to that of WT E18.5 and P4 lungs (Figure 4E–F). Taken together, our modified renal capsule allograft model for lung organ culture supports both the saccular development and vascular formation and would offer great advantage for the study of the molecular basis regulating late stages of lung development.

### Dorsomorphin rescues the atelectasis of *Fstl1^−/−^* lung in the renal capsule allografts model

**Figure 5:**
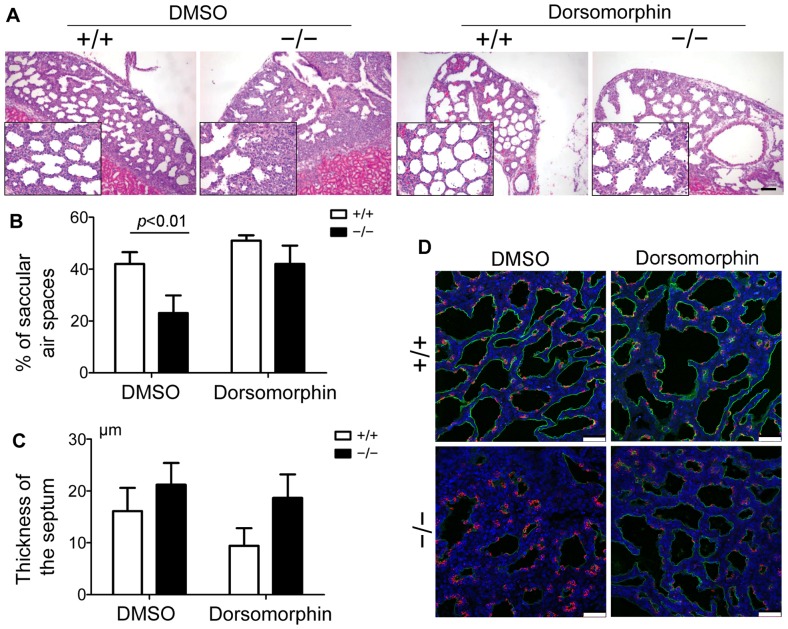
Reducing Bmp4 signaling activity rescued atelectasis phenotype of *Fstl1^−/−^* lung allografts. Diced E15.5 WT or *Fstl1^−/−^* lung allografts were implanted underneath the renal capsule of mice with or without Dorsomorphin treatment and cultured for 3 days. (A) Dorsomorphin increased saccular dilation of *Fstl1^−/−^* lung allografts. H&E staining of allograft sections shown at low and high (inserts) magnification. After Dorsomorphin treatment, *Fstl1^−/−^* lung allografts had more dilated sacs. Scale bar, 200 μm. (B) Quantification of saccular air spaces at the periphery in renal allografts from each experimental group (n = 3). The graph represents the mean ± SEM of three independent experiments. (C) Quantification of thickness of the septum at the periphery in renal allografts from each experimental group (n = 3). The graph represents the mean ± SEM of three independent experiments. (D) Dorsomorphin increased T1α staining but decreased pro SP-C staining in *Fstl1^−/−^* lung allografts. Sections of WT or *Fstl1^−/−^* lung allografts were immunostained with Pro SP-C and T1α antibodies. Red color: Pro-SPC; Green color: T1 α; blue color: DAPI. Scale bar, 50 μm.

To verify the above renal capsule allograft model, we implanted E15.5 WT or *Fstl1^−/−^* diced lung under the renal subcapsular of FVB/N mice and cultured for 3 days. No significant differences were detected between the WT and *Fstl1^−/−^* embryos at E15.5, but dramatic differences were visible from E17.5 onward [25]. Histological analysis revealed the collapsed phenotype in D3 *Fstl1^−/−^* allografts, as indicated by the thickened hypercellular intersaccular septa and a 45% reduction in air sac spaces than that of WT controls (Figure 5A–C). Immunofluorescence examination with antibodies specific for AEC-II cells (pro SP-C) or AEC-I cells (T1α) showed the impaired epithelial differentiation in immature saccular in *Fstl1^−/−^* allografts, as indicated by the increased pro SP-C staining and decreased T1α staining when compared to WT control (Figure 5D). Findings are in agreement with the abnormal phenotype of E18.5 *Fstl1^−/−^* lung in utero [25], confirming that the hypoplastic *Fstl1^−/−^* allografts in the renal capsule model follow the development pattern as if they remained in utero.

To further verify this model, we pretreated the host mice with an intraperitoneal injection of dorsomorphin, a small molecule inhibitor of Bmp signaling [33], to reduce the increased activity of Bmp4 signaling in *Fstl1^−/−^* allografts. After 3 days under renal capsule, dorsomorphin-treated *Fstl1^−/−^* allografts displayed more evenly dilated sacs with thinner septa, indicating the atelectatic defect due to the deletion of *Fstl1* was partially rescued (Figure 5A–C). Moreover, dorsomorphin-treated *Fstl1^−/−^* allografts had dispersed pro SP-C staining and increased T1α staining (Figure 5D), confirming that epithelial differentiation was partially rescued. Our data support that Fstl1 is a Bmp4 signaling antagonist in controlling lung development [25]. This is also in agreement with our previously study in which reduction of Bmp signaling activity by Bmp antagonist Noggin rescues pulmonary atelectasis of *Fstl1^−/−^* allografts. Therefore, our diced lung allograft under renal capsule provides a useful experimental model for the functional study of Fstl1 during the late stages of lung development.

### Impaired elastin deposition in *Fstl1^−/−^* lung allografts

**Figure 6:**
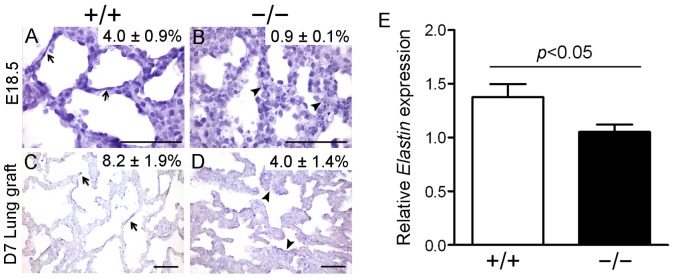
Impaired elastin deposition in E18.5 *Fstl1^−/−^* lungs and D7 *Fstl1^−/−^* lung allografts. Sections of fetal lung tissues and lung allografts were Weigert stained to visualize the deposition of elastic fibers. (A) E18.5 WT lung tissue, (B) E18.5 *Fstl1^−/−^* lung tissue, (C) day 7 WT lung allograft, (D) day 7 *Fstl1^−/−^* lung allograft. Elastic fibers were localized along the respiratory saccules and at the tip of the growing septa (arrows) in E18.5 WT lung (A) and D7 WT lung allografts (C). Elastin fibers could be barely seen in E18.5 *Fstl1^−/−^* lung (B, arrowheads) and showed a disorganized deposition (D, arrowheads) in D7 *Fstl1^−/−^* lung allograft when compared to their WT controls. Quantitative image analysis showed that *Fstl1* deletion reduced the elastin fiber density, expressed as a percentage of lung parenchyma, in *Fstl1^−/−^* lungs and allografts. Data are shown in the top right corner of each diagram (*P*<0.05). Scale bar, 50 μm. (E) Elastin production was decreased in E18.5 *Fstl1^−/−^* lungs using qRT-PCR analysis (n = 6). The graph represents the mean ± SEM of three independent experiments.

Elastin is an important extracellular matrix component of the lung. Irrespective of its role in alveolar septum integrity, elastin is essential for lung development. The deposition of elastin in the walls of terminal air sacs before birth provides sufficient elasticity in these structures to allow for efficient breathing after birth, while its deposition in alveolar secondary septa after P4 provides a critical driving force of alveologenesis [3], [4], [34]. To determine the role of Fstl1 on the elastin production during alveolar development, E18.5 lungs were Weigert stained to visualize elastic fibers [35]. As shown in Figure 6A, E18.5 WT lungs displayed continuous elastin fibers in the adjacent mesenchyme surrounding the air sacs. In contrast, E18.5 *Fstl1^−/−^* lungs displayed spotted elastin staining and significant less elastic fiber density (Figure 6B). Furthermore, we found a reduction in the transcription level of *Elastin* in E18.5 lung tissues of *Fstl1* knockout mice by quantitative RT-PCR analysis (Figure 6E). These data reveal that Fstl1 plays an important role in regulating the expression and deposition in primary septae and the cause of postnatal death of *Fstl1^−/−^* pups.

To determine whether Fstl1 is also involved in the elastin deposition in alveolar septae during the late saccular formation, we examined the diced lung allografts underneath the renal capsule because of the postnatal death of *Fstl1^−/−^* pups. Weigert staining of D7 WT lung allografts, whose developmental status is equal to P4 lungs as we suggested above, showed a remarkable elastin deposition in tips of the ridges of growing secondary septa (Figure 6C), indicating an intimate association with the initiation of alveolar septation [1]. Notably, compared to WT, there were irregular distributed elastic fibers with less density in the walls of distal sacs of D7 *Fstl1^−/−^* lung allografts (Figure 6D). Thus, Fstl1 is required for the proper positioning of elastin fibers and is involved in septa formation of alveoli.

## Discussion

During the late stages of lung development, embryonic lung matures into a functional gas-exchange unit, by developing numerous alveoli with septation of terminal air sacs [1]. In addition to genetic manipulation of animal models, advances of *in vitro* culture techniques can increase our understanding of the whole process of alveolar development. In this study, we have modified the allografts model by grafting E15.5 diced lung underneath the renal capsule of syngeneic mice (FVB/N background). Examination of developmental processes and Bmp signaling modulation in these diced lung allografts support the efficiency of this model for *ex vivo* culture of lung organs. Our studies using this robust model suggest a role of Fstl1 in elastin expression and deposition in walls of air sacs and subsequent alveolar septation.

Various allograft or xenograft models have been developed using immunocompromised mice as hosts for the study of tumor growth, pathogen infection, as well as organ development including lung [36], [37], [38]. Whole fetal mouse lung has been allografted subcutaneously into immunodeficient mice by Schwarz et al [39] and Chinoy et al. [40]. However, it takes up to 14 days for the E14.5 lung rudiments to reach the early saccular stage. To overcome the retardation of lung development in the subcutaneous model, the lung renal capsule allograft model has been developed [30], [41], [42], [43]. For example, individual whole E12.5 lung or dissociated E14.5 lung cells were placed underneath the renal capsule of immunocompromised mice and the grafts went through the canalicular and saccular stages at a pace similar to in utero development and with extensive blood vessel development [30], [42]. The comparison studies with implanted human fetal lung tissues in the renal subcapsular or dorsal subcutaneous space of immunodeficient mice show that the subcapsular region of the kidney provides a better site for the lung grafts to follow the correct remodeling process [41], [43]. The reason may be that the renal subcapsular region could accommodate tissues of a range of sizes and sources with a ready and functional blood supply [44].

In this study, we grafted E15.5 diced lung underneath the renal capsule of syngeneic mice for 3 and 7 days. In agreement with the whole fetal lung allografts, E15.5 diced lung allografts succeeded to progress from the canalicular to saccular stages in a manner similar to that in utero. Our results support that there is a pool of multipotent progenitor cells in the tips of the lung during pseudoglandular stage and these progenitors follow the intrinsic development potential even their environment has been changed [1]. Moreover, E15.5 diced lungs from *Fstl1^−/−^* mice were also grafted under the renal capsule. Not surprisingly, we found that they followed their intrinsic development potential [25] as indicated by the smaller air sacs, thickened intersaccular walls and retarded distal differentiation of lung epithelial cells. As Fstl1 is a secreted protein, our results suggest that the lung graft model can be used to distinguish the phenotypes caused by local and systemic effect due to loss of Fstl1 function. Our data also suggest that syngeneic mice have less effect on the developmental pattern of diced lung allografts, indicating a more convenient host for the renal capsule model. Furthermore, the intraperitoneal injected Bmp4 signaling inhibitor dorsomorphin could regulate the Bmp4 signaling activity in *Fstl1^−/−^* allografts and rescue their phenotype of pulmonary atelectasis, suggesting that vessels developed in lung allografts may also connect to the vasculature of the host immunocompromised mice, and that our diced lung allograft under renal capsule provides a useful experimental model for the functional study of Fstl1 during the late stages of lung development. However, when the diced E15.5 lung allografts were leaved under the renal capsule for 14 days, we observed the continued growth of allografts with the formation of “large alveoli” but without the formation of secondary septation, the process of typical secondary alveolarization. Early lung development occurs in a relative hypoxic environment, which is beneficial for lung branching morphogenesis [45], [46], but alveolarization after birth is stimulated by “breathing” movements and influenced by oxygen tensions and mechanical forces which the renal capsule can't provide [47], [48], [49]. This may explain the abnormal D14 lung allografts. Taken together, our diced lung renal capsular allograft model is a robust *ex vivo* organ culture model for studying saccular stage of lung development.

We further observed the disorganized elastic fibers in lung parenchyma of E18.5 *Fstl1^−/−^* mice and in alveolar septation of D7 diced *Fstl1^−/−^* lung allografts, indicating a role of Fstl1 essential for elastogenesis during lung alveolization. Defective elastin synthesis and deposition may contribute to the delayed saccular maturation and alveolar septation [3], [4], [34]. These data suggest that this diced lung renal allograft model can provide us with another way to study the late lung development in gene knockout mice which die in utero. Elastin production is the index of functional maturity of certain mesenchymal-derived cells and regulated by Pdgfα, glucocorticoid, CTGF and other signalings [4], [34], [50], [51]. The exact mechanism whereby Fstl1 in mesenchymal cells regulates the elastin secretion is actively pursued in our laboratories.

Taken together, the diced lung allograft underneath the renal capsule is an excellent and efficient model to study the developing potential of the distal progenitor populations and can help us to understand the molecular mechanisms of lung development before birth in normal versus hypoplastic lungs.

## Materials and Methods

### Ethics Statement

All animal experiments were approved by the Animal Care and Use Committee at Nankai University. Pregnant mouse was sacrificed by cervical dislocation 15.5 days post-coitus, the uterus was removed and the embryos were isolated quickly. After decapitation of the embryos, the lungs were rapidly removed and used for subsequent experiments. The kidney surgery was performed under sodium pentobarbital anesthesia, and all efforts were made to minimize suffering.

### Mouse Strains

*Fstl1^+/−^* mice were generated as previously described [25]. Intercross of *Fstl1^+/−^* mice that had been backcrossed onto the FVB/N background for at least 12 generations produced null mutant mice (*Fstl1^−/−^*).

### *In vitro* culture of E15.5 lung explants

Saccular explants culture was performed as described [52]. Brieﬂy, E15.5 WT fetal mouse lungs were isolated and dissected free of surrounding structures. The lung tissues were minced into 0.5–1 mm^3^ cubes and cultured on an air–liquid interface using permeable supports (Transwell, 0.4-μm pore size; Corning) and serum-free DMEM. Explants were cultured at 37°C in 95% air/5% CO_2_ for up to 3 days.

### Diced embryonic lung allografts under the renal capsule

The E15.5 WT and *Fstl1^−/−^* lung tissues were diced into 0.5–1 mm^3^ cubes and implanted under renal capsule according to the protocol on line by Brody J, Young P and Cunha G (http://mammary.nih.gov/tools/mousework/Cunha001/index.html). Brieﬂy, the female FVB/N mouse was weighed and anesthetized by sodium pentobarbital. Then a small incision was made in the body wall just slightly longer than the long axis of the kidney. The kidney was removed from the body cavity gently and was cut with a 2 to 4 mm incision in the capsule with fine spring-loaded scissors. The cut edge of the kidney capsule was lifted with the pair of fine forceps, and the diced lung tissues were inserted into the pocket under the capsule using the polished glass pipette. After implantation, the kidney was placed back into its position. In the rescue experiments, host mice were pretreated with an intraperitoneal injection of dorsomorphin (5 mg/kg body weight, i.p.) or DMSO vehicle 4 hours before and 1 day after the surgery for WT and *Fstl1^−/−^* lung allografts.

### Histological and immunohistochemistry analysis of the murine lung transplants and allografts

Lung transplants or allografts harvested at independent timepoints separately were fixed in 4% paraformaldehyde, dehydrated, and paraffin embedded. Fixed tissue was sectioned at 5-µm intervals, stained with hematoxylin and eosin for lung morphology or Weigert staining to visualize elastic fibers [35]. Then the sections were examined at the light-microscopic level for structural indexes of lung maturation. Mean saccular air spaces, thickness of the septum in lung or the relative amount and distribution of elastin in lung parenchyma was determined by computer-assisted morphometry with Image Pro Plus 6.0 software (Media Cybernetics) [25], [53]. For immunohistochemical analysis, the sections were hydrated, heated in 10 mM citrate buffer (pH 6.0) and blocked with 5% of normal serum. The sections were then incubated with primary antibodies at 4°C overnight. After extensive wash, samples were incubated with Cy3-labeled or Cy5-labeled secondary antibody (Jackson ImmunoResearch Laboratories, Inc., West Grove, PA, USA) for 1 hour. DAPI VECTASHIELD solution (Vector Laboratories Inc., Burlingame, CA, USA) was used as a mounting medium. The sections were then photomicrographed on a TCS SP5 confocal microscope (Leica). The Antibodies used for IHC were: pro-SP-C (Abcam, Cambridge, MA, USA); and T1α (also known as Pdpn) (Santa Cruz Biotechnology Inc., Santa Cruz, CA, USA); Endomucin (eBioscience Inc., San Diego, CA, USA).

### Quantitative RT-PCR analysis

Total RNA was isolated with Trizol (Invitrogen, Carlsbad, CA), cleaned with the RNeasy Mini Kit (Qiagen, Valencia, CA) and the DNA-free kit (Ambion, Austin, TX), then quantitative (q) RT-PCR was performed using FastStart Universal SYBR Green Master mix (Roche Diagnostics GmbH, Mannheim Germany) according to the manufacturer's protocols. Gene expressions were measured relative to the endogenous reference gene, mouse *β-actin*, using the comparative CT method described previously [54]. *Elastin* specific primer set was used for amplification: *Elastin* (NM_007925), forward 5′-CAG AGC TCC TCC TCC TCC TC-3′ and reverse 5′- CTT GCT CAA CCT CCT CCA TC -3′.

### Statistical Analysis

Data are expressed as the mean ± SEM. Differences in measured variables between experimental and control group were assessed by using Student's *t* tests. Results were considered statistically significant at *P*<0.05.
